# Evaluation of the nanotube intrinsic resistance across the tip-carbon nanotube-metal substrate junction by Atomic Force Microscopy

**DOI:** 10.1186/1556-276X-6-335

**Published:** 2011-04-14

**Authors:** Maguy Dominiczak, Larissa Otubo, David Alamarguy, Frédéric Houzé, Sebastian Volz, Sophie Noël, Jinbo Bai

**Affiliations:** 1 Lab. MSSMat, UMR CNRS 8579, Ecole Centrale Paris, Grande Voie des Vignes, Châtenay-Malabry 92290, France; 2 LGEP, UMR CNRS-SUPELEC 8507, Universités Paris Sud XI et UPMC, 11 rue Joliot-Curie, Plateau de Moulon, Gif-sur-Yvette 91192, France; 3 Lab. EM2C, UPR CNRS 288, Ecole Centrale Paris, Grande Voie des Vignes, Châtenay-Malabry 92295, France

## Abstract

Using an atomic force microscope (AFM) at a controlled contact force, we report the electrical signal response of multi-walled carbon nanotubes (MWCNTs) disposed on a golden thin film. In this investigation, we highlight first the theoretical calculation of the contact resistance between two types of conductive tips (metal-coated and doped diamond-coated), individual MWCNTs and golden substrate. We also propose a circuit analysis model to schematize the «tip-CNT-substrate» junction by means of a series-parallel resistance network. We estimate the contact resistance *R *of each contribution of the junction such as *R*_tip-CNT_, *R*_CNT-substrate _and *R*_tip-substrate _by using the Sharvin resistance model. Our final objective is thus to deduce the CNT intrinsic radial resistance taking into account the calculated electrical resistance values with the global resistance measured experimentally. An unwished electrochemical phenomenon at the tip apex has also been evidenced by performing measurements at different bias voltages with diamond tips. For negative tip-substrate bias, a systematic degradation in color and contrast of the electrical cartography occurs, consisting of an important and non-reversible increase of the measured resistance. This effect is attributed to the oxidation of some amorphous carbon areas scattered over the diamond layer covering the tip. For a direct polarization, the CNT and substrate surface can in turn be modified by an oxidation mechanism.

## Introduction

Since the official publication of the carbon nanotubes (CNTs) images during the period of 1950 to 1990 [1], these allotropes have become very promising candidates for various applications because of their outstanding electrical, mechanical and thermal characteristics. They have competed for a high-level development in many fields such as nanoelectronic devices and nanoelectromechanical technologies: for example field-effect transistors (FETs), nano electro mechanical systems (NEMS), nano random access memories (NRAMs), nanoelectronic logic circuits and also nanomotors based on semiconducting CNTs [2-6]. A single-walled carbon nanotube (SWCNT) may behave either as a conductor or as a semiconductor. Electrical properties of nanotube are highly dependant on their atomic structure [7]; for example the conductivity of SWCNTs depends on their chirality in the honeycomb lattice structure of graphene and their diameter [8] as well as the electrical contact nature. CNTs have gained a renewed interest in the past few years, owing to their high conductance and high electron mobility [9,10]. The strength of the sp^2 ^(C-C) covalent hybridization bonds brings carbon nanotubes noteworthy mechanical properties too [11-13]. Multi-walled carbon nanotubes (MWCNTs) consist of several concentric SWCNTs held together by Van der Waals interactions. The spacing between two consecutive graphene sheets is about of 3.4 Å and the intershell conduction is governed by the electron hopping mechanism, which depends on the overlap of the carbon π-orbitals between neighboring layers. MWCNTs present an anisotropic metallic behaviour [14] because of the stacking of the graphite sheets. Multi-walled carbon nanotubes have the advantage to be easier to connect and give contact resistances lower than SWCNTs ones. Indeed, the contact resistance between a SWCNT and a metal contact cannot be lower than a few kΩ [15-18]. In the literature, researches based on the electrical contact resistance on MWCNTs have been previously published [19]. Lan et al. studied the electrical contact between an individual MWCNT and a deposited metallic film. The contact resistance is modelled as a sequence of resistors that tie the CNT along its entire length. An uncovered length of the CNT bridges the gap between the two separate Ti/Au electrodes on which is applied a bias voltage.

In this paper, investigations are focused by another approach than [19], on the study of the radial contact resistance between a conductive tip and a single MWCNT, then between this CNT and a metal substrate. By means of conductive probe atomic force microscopy (CP-AFM), we characterize at room temperature CNTs by electrical imaging in order to measure their local resistance. The key requirements allowing to deduce the CNT intrinsic radial resistance are discussed by proposing a resistance model for the «tip-CNT-substrate» junction. The contact resistance *R *of each contribution as *R*_tip-CNT_, *R*_CNT-substrate _and *R*_tip-substrate _can be calculated by combining the Hertz's mechanical formula of contact area and the Sharvin's ballistic resistance model [20,21]. The functionalization of CNTs with gold nanoparticles (AuNPs) is also investigated as a possible mean to improve their electrical conductivity. Finally, the contiguous question of local modification of the CNT and substrate surface is raised after operating at various bias voltages with diamond tips.

## Methods and materials

### Elaboration and purification of the MWCNTs

Carbon nanotubes have been elaborated by chemical vapour deposition (CVD) in a tubular furnace through a reactor (quartz tube) under a mixture of argon, hydrogen and acetylene gas. This production method can fabricate MWCNTs in large quantity. Observations in transmission electron microscopy (TEM) showed an entanglement of synthesized MWCNTs, which grow from catalysts in different geometrical configurations as straight or helical shapes. The catalytic activity realized with a mixture of ferrocene and xylene (as carbon source) was obtained by heating up to 750°C for 10 min. By thermal oxidation, the amorphous carbon structure was eliminated at 300°C during 1 h 30 min in air for purification. CNTs were then mixed with a nitric acid treatment for removing the metallic catalyst impurities [22,23]. By acid treatment, it has been observed at optical microscope with Surf substrate («Nanolane» manufacturer, France) that the CNTs were best-purified. The as-prepared solution was uniformly dispersed by sonication during 2 min to separate the aggregations and then filtered. These CNTs were then ultrasonically diluted with DMF (*N*,*N*-dimethylformamide) solvent for 4 min, before AuNPs grafting for some of them (see further the first section of Results and discussion).

### Au surface preparation

The golden substrates used for the study were 5 × 5 mm^2 ^coupons obtained from a Si wafer covered with a 10-nm Cr adhesion layer and an Au layer of about 200 nm by physical vapour deposition (PVD). Gold has been considered as a reference material surface to investigate the electrical transport properties of the MWCNTs. The dispersion solution containing CNTs was then deposited onto these substrates.

### Atomic force microscope

For all the experiments reported below, we used a D.I. Nanoscope IIIa Multimode AFM equipment associated with a LGEP home-made system called 'Resiscope' [24] dedicated to the local electrical resistance measurement. The as-prepared substrates are then fixed with silver paint on the steel sample holder placed on the AFM piezoelectric actuator. The surface morphology of the CNTs was imaged at room temperature (300 K), in the standard contact mode. We used two types of commercial conductive probes: (i) *N*-doped silicon probes coated with a P-doped diamond layer and (ii) Pt/Ir coated Si probes, both of them with a nominal k spring constant in the range 1 to 5 N/m («Veeco Probes» manufacturer, USA). The average curvature radius (*r*_t_) of the diamond tip is of about 150 nm and the Pr/Ir tip one of 20 nm. Topography and resistance cartographies were simultaneously recorded, applying a DC bias between the substrate and the tip. The Resiscope range covers ten decades from 10^2 ^to 10^12 ^Ω. For a given zone, successive scans at different bias voltages were performed in order to determine the sensitivity to this parameter.

## Results and discussion

### Contact resistance measurement methods

#### Comparison between Pt/Ir and diamond tips

**Figure 1:**
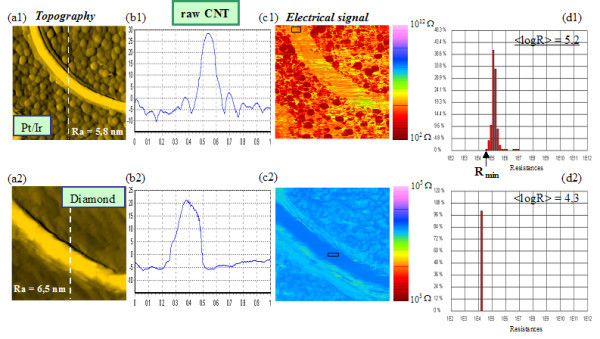
**(a1, a2) AFM topographic images (1 × 1 μm^2^) of a 'raw' CNT obtained with a Pt/Ir and a diamond tip, respectively; (b1, b2) CNT height profile along dotted lines; (c1, c2) corresponding electrical maps; (d1, d2) distribution histograms of resistance values measured in the region marked out by a rectangle on the CNT**. The cantilever load-force was about 16 to 80 nN, respectively, for *k *= 1 to 5 N/m. *V*_tip-sample _= +1 V.

Figures 1a1 and 1a2 show typical topographic AFM images (1 × 1 μm^2^) of a MWCNT obtained with a Pt/Ir tip and a diamond tip, respectively; b1 and b2 show typical cross-sections along the dotted lines; c1 and c2 show the associated electrical cartographies of the CNT (+1 V bias) and d1 and d2 show the corresponding distribution histogram of the local resistances *R *measured within a rectangle selected along the CNT. The CNT diameter can be estimated by considering the height profile in the topography images. With the diamond tip, the CNT has an apparent width larger than its height in the topographic view as well as in the resistance image. However, the image width obtained with a Pt/Ir tip gives a value closer to the real CNT diameter. The contact area of the Pt/Ir tip is very small compared to the diamond tip, which does not have a perfect tetrahedral geometry due to the coating morphology. One can be convinced looking at microscopy images on the manufacturer's website [25]. The MWCNTs observed by high resolution TEM have an average of 30 to 40 walls with an external diameter in the 15 to 40 nm range. These values are in accordance with the AFM observations, since from the scanned CNTs in Figure 1 nominal diameter can be estimated between 20 and 35 nm. The average roughness Ra on the substrate surface is given on the topographic images. For more clarity, the topography images were fitted in plane and then the structures were raised using an arithmetic mean in a 5 × 5 matrix.

Electrical images from diamond tip were re-scaled between 10^3 ^and 10^5 ^Ω in order to improve the electrical contrast between the CNT and the substrate (when using the full scale between 10^2 ^and 10^12 ^Ω, no difference can be observed). It can be deduced from the electrical image that the CNT on the whole scanned area presents a homogeneous conductivity, indicating a good electrical contact with the substrate. This can imply a high carrier density, which is controlled by hole conduction near to Fermi level, given that MWCNTs are hole-doped in air [26]. The barrier for electrons is high because of the pinning of the Fermi level close to the valence band maximum at the CNT-substrate interface [27]. In Figure 1d1 and 1d2, on the left side of the distribution histogram is shown the minimum electrical resistance *R*_min _measured within the black rectangle selected from the electrical image. The *R*_min _value relative only to the substrate (image not shown here) corresponds to the intrinsic tip resistance: *R*_Pt/Ir-tip _~ 10^2 ^Ω and *R*_diamond-tip _~ 10^4 ^Ω. From electrical images, we calculated the average of *Log*(*R*) values along the CNT length (Table 1). We have observed that <Log(R)> is constant along the CNT, so we can deduce a good ohmic contact quality between the CNT and the gold substrate.

**Table 1:** Average of the whole *<Log(R)>*values of several rectangles selected along the CNT length on raw CNT and CNT functionalized with AuNPs (direct bias +1 V).

Tip	Raw CNT	CNT-AuNPs	Substrate
Pt/Ir			
<Log(R)>	5.3	5.7	4
*R*_Total _(Ω)	2 × 10^5^	5 × 10^5^	1 × 10^4^
Diamond			
<Log(R)>	4.3	4.5	4.3
*R*_Total _(Ω)	2 × 10^4^	3.2 × 10^4^	2 × 10^4^

On the other hand, *R*_Total _must be measured with a Pt/Ir tip because *R*_Pt/Ir-tip _is very low compared to *R*_diamond-tip_. Moreover, we have pointed out the resistance measured with a Pt/Ir tip is higher (one decade) on the CNT than on the substrate. Accordingly, there is no resistance filtering with a Pt/Ir tip, but it is not true with a diamond tip (see Table 1). *R *diamond-coated tip and CNT is approximately similar to *R *diamond-coated tip and substrate. We do not distinguish them plainly because the intrinsic diamond tip resistance brings a very important and non-negligible contribution across the «tip-CNT-substrate» junction. Hence, we consider that measurements with a diamond tip are not as reliable as the ones with a Pt/Ir tip. We also noticed that the resistance value is nearly one decade higher on the CNT when measured with a Pt/Ir tip than when obtained with a diamond one. A larger contact area, at a given force, for the diamond tip apex [25] could explain why *R*_diamond-CNT _is lower than *R*_Pt/Ir-CNT_.

**Figure 2:**
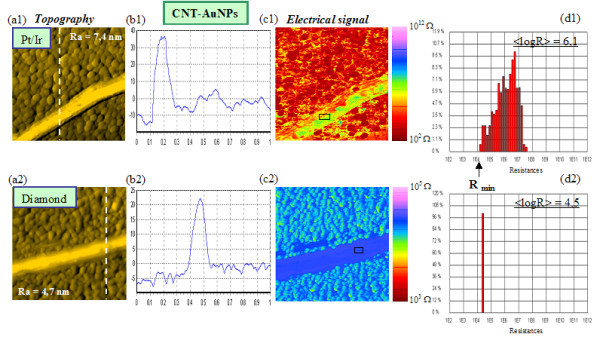
**(a1, a2) AFM topographic images (1 × 1 μm^2^) of a CNT functionalized with AuNPs, obtained with a Pt/Ir and a diamond tip, respectively; (b1, b2) CNT height profile along dotted lines; (c1, c2) corresponding electrical maps; (d1, d2) distribution histograms of resistance values measured in the region marked out by a rectangle on the CNT**. Same experimental parameters as for Figure 1.

Besides these measurements on 'raw' CNTs, a series of tests on CNTs functionalized with AuNPs was also carried out in order to see if electrical properties of the CNTs could be modified. AuNPs are produced with a few drops of DDAB (didodecyldimethylammonium bromide) type organic molecule, introduced to reticulate the nanoparticles to CNTs by covalent bonds (micellar system). The particle size is about 5 nm. Figures 2a1 and 2a2 show topography views of a CNT with an AuNPs attachment for a conductive Pt/Ir tip and a diamond tip, respectively, b1 and b2 the corresponding cross-sections along dotted lines, c1 and c2 the associated resistance images (always under +1 V bias) and d1 and d2 typical distribution histograms of the local resistances. The a2 topography obtained with a diamond tip has a better resolution, showing individual gold grains, than the one obtained on raw CNT. We did not measure any resistance reduction of the MWCNTs with a gold nanoparticle functionalization (Table 1). Grafting of AuNPs may not be uniformly distributed and disposed in large quantity along MWCNTs. We think that the AuNPs are not enough numerous to induce a modification of the global electrical properties with CP-AFM. One of the main problems of the measurement with the AFM tip is that an individual CNT can slide under the tip pressure [28]. This is why the CNT position has sometimes changed between two successive pictures and the observed area can be rid of nanoparticles due to tip scanning. Hence, CNTs with metal nanoparticles in our situation were not found to show an improved conductivity by CP-AFM measurements, but they allowed us to check the reproducibility of the results when varying voltage bias as will be seen further (see section 'DC voltage effects').

#### «Tip-CNT-substrate» junction analysis model

**Figure 3:**
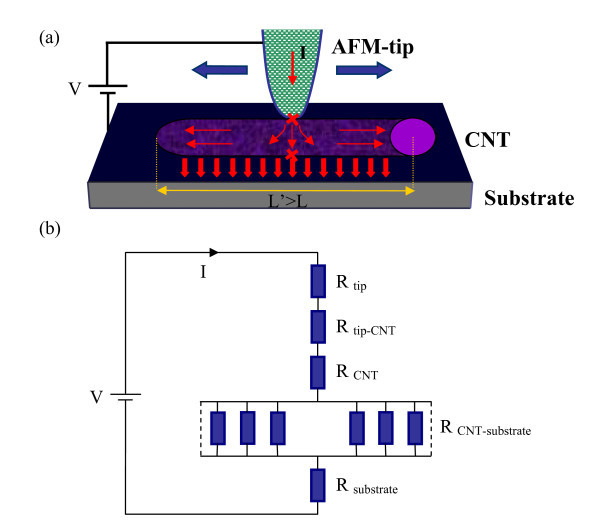
**(a) Schematic view of the AFM tip and the «tip-CNT-substrate» junction**. A bias voltage *V *is applied between the tip and the substrate, the arrows represent the direction of the current lines. **(b) Series-parallel resistance network corresponding to setup scheme**.

The current conduction of the «tip-CNT-substrate» junction is mainly realized along the CNT radial direction (see Figure 3a). A schematic model of the resistance network for the nanocontact between the tip and the sample can be imagined in the following way with series-parallel resistances (Figure 3b), which is consistent with the previously published results [19,29]. The global resistance measured can thus be considered as the sum of several contributions:(1)

The bias voltage *V *applied between the tip and the substrate, supplies the two junctions in series tip-CNT and CNT-substrate. The CNT-substrate interface is supposed to be formed by a number of elementary contacts at the top of roughness hills and therefore simulated by a parallel resistance network. The current trajectory across the MWCNT is anisotropic (Figure 3a).

#### DC voltage effects

**Figure 4:**
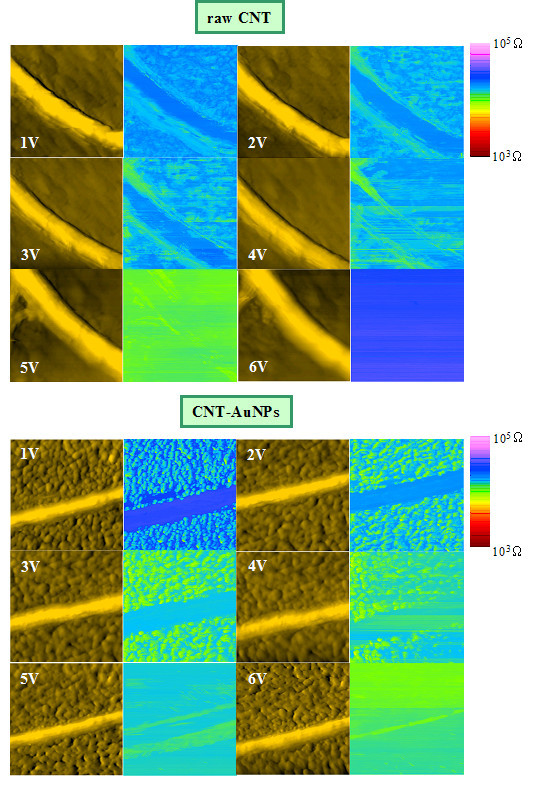
**Topography (left) and resistance maps (right) of raw CNTs and CNTs with AuNPs using a diamond tip for various polarizations in the range 1 to 6 V (scan size of 1 × 1 μm^2^)**.

**Figure 5:**
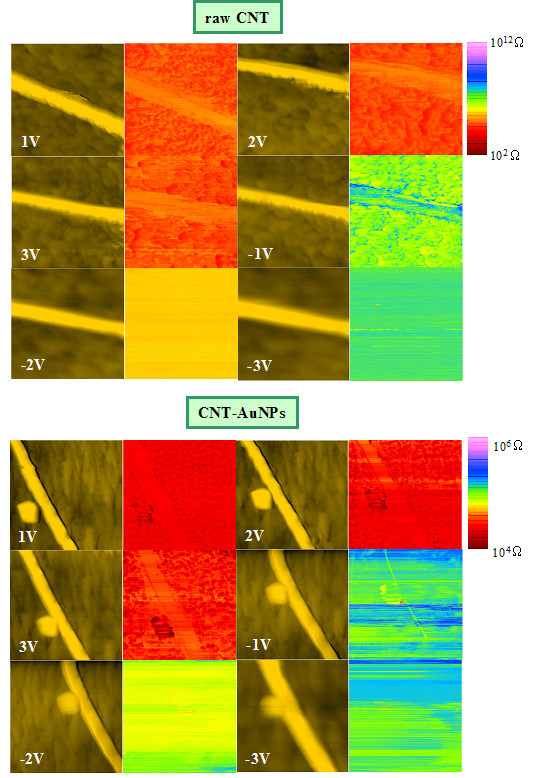
**Topography (left) and resistance maps (right) of raw CNTs and CNTs with AuNPs using a diamond tip for various polarizations between ±3 V**. The scan length is 1 μm. For the electrical images obtained on CNTs with AuNPs, the resistance scale is plotted in the range of 10^4 ^to 10^6 ^Ω to enhance the contrast.

In Figures 4 and 5 are represented series of AFM/Resiscope pictures of the raw CNTs and CNTs functionalized with AuNPs acquired with a diamond tip under several polarizations: from 1 up to 6 V in Figure 4 and from +1 to +3 V and -1 to -3 V in Figure 5. For the highest bias values, we can see a noticeable loss in resolution on the AFM images obtained on CNTs with AuNPs in Figure 4. The individual gold grains are not so clearly visible, but as expected the mean resistance calculated over the electrical cartography decreases as the bias is increased, except in the case of 6 V for raw CNT. The comparison of the resistance images in Figure 5 in direct and reverse polarization allows us to conclude that the current-voltage characteristic should not be symmetrical. To take into account a better approach of the conduction mechanism with the diamond tip, we adapt our resistance model (see Figure 3b) by introducing the additional contribution of a Schottky diode between the *R*_tip-CNT _contact junction (resistance dominating in the circuit as we will see it in section of 'Sharvin's model') and *R*_CNT_, so that the diode allows the current to flow in a single direction. It was reported that the charge transport in CNTs is controlled by the Schottky barriers that forms the metal-CNT junction, the nature and geometry of this contact can strongly modify the electrical behaviour [30,31]. Let us bring up again the particularity of the results under reverse bias shown in Figure 5. The negative polarization seems to affect the tip coating. As AFM is operated in ambient air, a possible explanation could be that a local redox reaction occurs in the water meniscus at the tip apex [32], inducing an increase of the measured resistance. Such an effect could also induce on our samples a local surface modification, since the electrical contrast between the CNT and the substrate disappears between -2 and -3 V. Concerning the tip, a hypothesis could be the oxidation of small amorphous carbon domains scattered over the diamond coating of the tip for *V *< 0. Mahé et al. examined precisely the electrochemical reactivity effect on diamond electrodes covered with graphitic micro-domains [33]. As regards the astonishing result in direct polarization at 6 V (see Figure 4), a hypothesis could be the oxidation of amorphous carbon residues on the CNTs combined with a transfer of the oxidized material to the tip. This is corroborated by the experimental observation that the return to the initial state is difficult as if an irreversible phenomenon affected the tip surface, making it unusable. From 6 to 1 V (Figure 4) and -3 to 1 V (Figure 5), it was not possible to recover the initial resistance levels, even after several successive scans. For the following discussion only results with no oxidation suspicion will be considered.

### Theoretical calculations

In this paragraph, a rough model is proposed in order to estimate each contact resistance contribution in Equation 1. These contributions are related to the constriction of the current lines at the tip-CNT, CNT-substrate and tip-substrate interfaces, therefore two combined models are required: a mechanical model giving the contact area, and an electrical model physically adapted to this size allowing to calculate the resulting resistance.

#### Hertz theory

Hertz's mechanical theory of elastic contact was chosen for its simplicity and because for our AFM measurements quite low contact forces were applied. Analytical solutions have the following form:(2)

where *a *is the contact radius and *F *is the force exerted by the cantilever, given as spring constant multiplied by the tip static deflection Δ*Z*. As we used a cantilever with *k *ranging from 1 to 5 N/m, a force between 16 and 80 nN can be estimated. *E*_1_, *E*_2_, ν_1 _and ν_2 _are respectively, the Young's moduli and the Poisson's ratios of the different materials involved in the tip-CNT, CNT-substrate and tip-substrate junctions (Table 2). *r*_t _is an equivalent radius of the curvature taking into account the radii *r*_1 _and *r*_2 _of the contacting bodies. The maximum pressure at the centre of the contact area can be expressed as:(3)

**Table 2:** Mechanical and electrical parameters of the various materials used for the junction «tip-CNT-substrate», with *E*_*i *_(Young's moduli), ν_*i *_(Poisson's ratio's), *r*_t _(curvature radius), ρ (resistivity) and *l *(electron mean free path).

	***E***_**1 **_**(GPa)**	**ν**_**1**_	***E***_**2 **_**(GPa)**	**ν**_**2**_	***r***_**t **_**(nm)**	ρ (Ω m)	*l *(nm)
Au	78^*1*^	0.42^*1*^			90	2.35 × 10^-8*2*^	36^*2*^
MWCNT	10^*3*^	0.28^*4*^			12.5^*3*^	10^-6*5*^	80^*6*^
Diamond			1063^*7*^	0.1^*7*^	150^*8*^	4 × 10^-5^[25]	40
Pt/Ir			233.3^*9*^	0.368^*9*^	20^*8*^	2.35 × 10^-8*2*^	36^*2*^

^1^from ref. [37]

^2^from ref. [21]

^3^from ref. [[38] (φ_CNT _: 25 nm), [39-41]]

^4^from ref. [41]

^5^from ref. [42]

^6^from ref. [43-45]

^7^from ref. [46]

^8^from tip manufacturer

^9^from ref. [47] (we take account a ratio of Pt-20%Ir)

Numerical values for *a *and *p*_0 _at the various interfaces considered are listed in Table 3.

**Table 3:** Calculation of the different contact pressure and radii for the tip-CNT, CNT-substrate and tip-substrate junction.

	***a***_**(1 N/m) **_**(nm)**	***a***_**(5 N/m) **_**(nm)**	***Po***_**(1 N/m) **_**(MPa)**	***Po***_**(5 N/m) **_**(MPa)**
Diamond–CNT	1.8	3.1	2358	3975
Pt/Ir–CNT	2.1	3.5	1732	3118
CNT–substrate	2.4	4.1	1326	2272
Diamond–substrate	2.0	3.4	1910	3304
Pt/Ir–substrate	1.4	2.4	3898	6632

#### Sharvin's model and calculations of the junction contributions

Whatever the considered interface, the contact radius is found very small compared to the electron mean free path of materials (reported in Table 2). This case can be described by a ballistic transport model like Sharvin's one. From this model, the contact resistance is given by:(4)

where *ρ*_*i *_and *l*_*i *_denote the resistivity and electron mean free path of the two materials. Calculation results are then summarized in Table 4. For convenience, the relationship valid for most metals, ρ·*l*_(Au) _= 8.46 × 10^-16 ^Ω m^2 ^[21] is used in the Pt/Ir case. The CNT mean diameter is of 25 nm (see Table 2). The substrate resistance was considered as negligible. For *R*_diamond-CNT_, we considered a grain of about 10 nm in diameter at the apex of the diamond tip, for calculating *a*, consistent with the imperfect probe geometry revealed by manufacturer's microscopy image [25]. We then calculated the equivalent curvature radius *r*_t _of the tip apex (assumed spherical) in contact with the CNT (considered as cylindrical). The gold grains of the substrate have a typical diameter of about 180 nm (Table 2). The spacing between two consecutive gold grains is around 100 nm. Therefore, for a 5 μm (*L*) and 20 μm (*L'*) long CNT, we can estimate 50 and 200 contact points, represented in the model of Figure 3 as a network of 50 and 200 parallel resistances. From Table 4, we can see that *R*_Pt/Ir-CNT _is higher than *R*_Pt/Ir-Au _confirming that the substrate is more conducting than the CNT. *R*_Pt/Ir-Au _is lower than *R*_diamond-Au _as the diamond tip is less conductive than the Pt/Ir one (results coherent with those reported in Table 1). *R*_diamond-CNT _is superior to *R*_Pt/Ir-CNT _considering a single grain of 10 nm at the diamond tip apex. The electrical resistance of an individual MWCNT at room temperature must be of about 10 to 50 kΩ as pointed out by several authors [34-36] using a four-point probe method. We can then deduce from Equation 1 (see Tables 1 and 4, Pt/Ir tip) an estimated value of the CNT resistance of approx. 10^5 ^Ω. We consider that the values of *R*_Pt/Ir _and *R*_Pt/Ir-CNT _(Table 4) are negligible with respect to *R*_Total _(Table 1). In the future, complementary investigations involving a four-point-probe measurement technique could probably allow us to establish more precisely the resistance of individual CNTs.

**Table 4:** Results of the contact resistance calculations for each interface (see Equation 1).

	Digital multimeter	*k *(1 N/m)	*k *(5 N/m)	**Diamond**_**(1 N/m)**_	**Diamond**_**(5 N/m)**_	**Pt/Ir**_**(1 N/m)**_	**Pt/Ir**_**(5 N/m)**_
*R*_tip_				10^4 ^Ω	10^4 ^Ω	10^2 ^Ω	10^2 ^Ω
*R*_tip-CNT_				110 kΩ	37.1 kΩ	3.9 kΩ	1.4 kΩ
*R*_CNT-substrate_		3 kΩ	1 kΩ				
*R*_substrate_	0.1 Ω						
*R*_tip-substrate_				84.9 kΩ	29.4 kΩ	183.2 Ω	62.3 Ω
*R*_CNT_						10^5 ^Ω	10^5 ^Ω

## Conclusions

Conducting probe atomic force microscopy in ambient air was used to investigate the local electrical resistance of MWCNTs disposed on thin gold films. The whole setup can be considered as a «tip-CNT-substrate» junction. By imaging individual CNTs, we were able to deduce their intrinsic radial resistance from the global one measured experimentally and the electrical contact ones calculated across the junction *via *a series-parallel resistance network model. Using a conductive Pt/Ir tip, we found a high resistance value of about 10^5 ^Ω for a cantilever load-force of about 16 to 80 nN with our AFM setup. For an application in electronic devices, this suggests the need to reduce the contact resistance by applying a more important load and to optimize the CNTs functionalization. Through this study, parasitic phenomena were also evidenced with diamond tips for negative bias voltages as well as some high positive ones, causing an irreversible increase of the measured electrical resistance. This observation was attributed to the redox reactions at the tip and/or sample surface leading to a local surface modification of the CNTs and substrate.

## Abbreviations

AFM: atomic force microscope; AuNPs: gold nanoparticles; CNTs: carbon nanotubes; CP-AFM: conductive probe atomic force microscopy; CVD: chemical vapour deposition; FETs: field-effect transistors; MWCNTs: multi-walled carbon nanotubes; NEMS: nano electro mechanical systems; NRAMs: nano random access memories; PVD: physical vapour deposition; SWCNT: single-walled carbon nanotube; TEM: transmission electron microscopy.

## Competing interests

The authors declare that they have no competing interests.

## Authors' contributions

MD realized all the AFM measurements as well as all the theoretical calculations, participated in the design of the study, wrote the manuscript, and coordinator between all the participants. LO made all the nanotube samples. DA took part in the study and contributed to the article improvement. FH participated in the study and contributed to the article improvement. SV read the article. SN read the article. JB participated in the conception of the project and contributed to the article improvement. Manuscript read and approved by all the authors.
